# Glucose Uptake and Intracellular pH in a Mouse Model of Ductal Carcinoma *In situ* (DCIS) Suggests Metabolic Heterogeneity

**DOI:** 10.3389/fcell.2016.00093

**Published:** 2016-08-31

**Authors:** Rebecca C. Lobo, Neil E. Hubbard, Patrizia Damonte, Hidetoshi Mori, Zsófia Pénzváltó, Cindy Pham, Amanda L. Koehne, Aiza C. Go, Steve E. Anderson, Peter M. Cala, Alexander D. Borowsky

**Affiliations:** ^1^Center for Comparative Medicine, University of California at DavisDavis, CA, USA; ^2^Department of Human Physiology and Membrane Biology, University of California at DavisDavis, CA, USA; ^3^Department of Pathology, School of Medicine, University of California at DavisSacramento, CA, USA

**Keywords:** ductal carcinoma *in situ*, mouse mammary carcinoma model, tumor heterogeneity, glucose uptake, intracellular pH, proton export, tumor microenvironment

## Abstract

Mechanisms for the progression of ductal carcinoma *in situ* (DCIS) to invasive breast carcinoma remain unclear. Previously we showed that the transition to invasiveness in the mammary intraepithelial neoplastic outgrowth (MINO) model of DCIS does not correlate with its serial acquisition of genetic mutations. We hypothesized instead that progression to invasiveness depends on a change in the microenvironment and that precancer cells might create a more tumor-permissive microenvironment secondary to changes in glucose uptake and metabolism. Immunostaining for glucose transporter 1 (GLUT1) and the hypoxia marker carbonic anhydrase 9 (CAIX) in tumor, normal mammary gland and MINO (precancer) tissue showed differences in expression. The uptake of the fluorescent glucose analog dye, 2-[N-(7-nitrobenz-2-oxa-1,3-diazol-4-yl) amino]-2-deoxy-D-glucose (2-NBDG), reflected differences in the cellular distributions of glucose uptake in normal mammary epithelial cells (nMEC), MINO, and Met1 cancer cells, with a broad distribution in the MINO population. The intracellular pH (pH_i_) measured using the fluorescent ratio dye 2′,7′-bis(2-carboxyethyl)-5(6)-155 carboxyfluorescein (BCECF) revealed expected differences between normal and cancer cells (low and high, respectively), and a mixed distribution in the MINO cells, with a subset of cells in the MINO having an increased rate of acidification when proton efflux was inhibited. Invasive tumor cells had a more alkaline baseline pH_i_ with high rates of proton production coupled with higher rates of proton export, compared with nMEC. MINO cells displayed considerable variation in baseline pH_i_ that separated into two distinct populations: MINO high and MINO low. MINO high had a noticeably higher mean acidification rate compared with nMEC, but relatively high baseline pH_i_ similar to tumor cells. MINO low cells also had an increased acidification rate compared with nMEC, but with a more acidic pH_i_ similar to nMEC. These findings demonstrate that MINO is heterogeneous with respect to intracellular pH regulation which may be associated with an acidified regional microenvironment. A change in the pH of the microenvironment might contribute to a tumor-permissive or tumor-promoting progression. We are not aware of any previous work showing that a sub-population of cells in *in situ* precancer exhibits a higher than normal proton production and export rate.

## Introduction

Ductal carcinoma *in situ* (DCIS) accounts for 27% of all breast cancers diagnosed in women over 50 (DeSantis et al., 2014), and since the 1970's it has been accepted as the non-obligate precursor of invasive ductal carcinoma (Cowell et al., 2013). Consequently, the majority of patients diagnosed with DCIS are subjected to lumpectomy and radiation or mastectomy. Determining the relationship between DCIS and Invasive Breast Cancer (IBC) and developing biomarkers for distinguishing low and high risk DCIS could prevent unnecessary treatment, financial and emotional burdens for low-risk DCIS patients (Esserman et al., 2009).

We have developed a mouse model of DCIS, called the mammary intraepithelial neoplastic outgrowth or MINO model, to study aspects of the progression of precancer to invasion in multiple contexts. The MINO model is derived from the Polyoma virus middle-T (PyVmT) transgene mouse model and consists of the transplantation of MINO from transgenic mice, FVB/N-Tg(MMTV-PyVmT) on an FVB background, to syngeneic immune-intact FVB mice (Maglione et al., 2001, 2004). Both DCIS and MINO are heterogeneous in cell type and function compared with corresponding invasive carcinomas (Damonte et al., 2008; Cowell et al., 2013). We previously showed that the heterogeneity that arises in lesions in the MINO model originates from precancer “stem” cells that are capable of self-renewal and multi-lineage differentiation with a programmed progression to invasive cancer (Damonte et al., 2008). MINO-derived precancer and cancer showed no significant differences in the genomic or telomere stability, suggesting that mechanisms other than genetic alterations may be responsible for the progression to invasion in the MINO model (Damonte et al., 2008). Epigenetic changes within neoplastic cells could account for the lack of significant changes in the genetic code between MINO and tumor. However, promoter DNA methylation has not been found to be significantly different between DCIS and IBC tumors suggesting that methylation changes may be early events in carcinogenesis rather than essential events in the transition to invasive disease (Moelans et al., 2011; Verschuur-Maes et al., 2012). Another possibility is that the invasive capabilities of a tumor cell are influenced by the surrounding microenvironment. There is considerable evidence that intracellular and extracellular pH can alter malignant cell survival and invasion (Gatenby et al., 2006; Rofstad et al., 2006; Webb et al., 2011).

Tumor cells metabolize ~10-fold more glucose to lactate than normal cells under non-hypoxic conditions. This increase in aerobic glycolysis is known as the Warburg effect (Warburg, 1956). Upregulated glycolysis has significant consequences. Subsequent elevated proton production can lead to a regional acidic microenvironment (Stubbs et al., 2000; Kato et al., 2013). Exposure of normal cells to an acidic microenvironment results in cell death (Park et al., 1999). Tumor cells compensate for increased proton production via increased proton export, largely through upregulation of the activity of the sodium-hydrogen exchanger (NHE1), to maintain an optimal, more alkaline pH_i_ compared with normal cells (Spugnini et al., 2015). There has been speculation about the role of this metabolic switch in cancer progression, specifically that acidosis leads to a tumor-permissive microenvironment by creating a hostile environment for normal cells, where acid resistance in tumor cells constitutes a proliferative advantage (Gatenby and Gillies, 2004; Webb et al., 2011; Spugnini et al., 2015).

Aerobic glycolysis is often accompanied by increased glucose uptake (Groves et al., 2007). We have previously shown that MINO and MINO-derived tumors can be imaged *in vivo* with PET imaging using the fluorescently labeled glucose analog [^18^F]2-deoxy-2-fluoro-D-glucose (FDG) (Abbey et al., 2006). However, due to resolution limits with FDG-PET, uptake by individual cells within the MINO or tumor cannot be distinguished. Based on our observations that precancer “stem” cells exist within the MINO, display no genetic differences from the rest of the MINO, and that MINO tissue exhibits increased FDG uptake, we hypothesized that there might be a sub-set of cells within the MINO with increased glucose uptake and high rates of proton production and export that condition the extracellular environment and permit or promote cancer progression and tumor invasiveness.

## Materials and methods

### Animals

Young female FVB/NJ (Charles River, Wilmington, MA) mice were used to obtain both normal and MINO tissue. Tumors (no larger than 1 cm) were from transgenic PyVmT females that had developed mammary tumors. Mice were housed in a vivarium under NIH guidelines and all animal experiments followed protocols approved by the UC Davis Institutional Animal Care and Use Committee (IACUC).

### Cell culture

The Met1 tumor cell line was developed previously in our laboratory (Borowsky et al., 2005). Briefly, Met1 was derived from mammary carcinomas in FVB/N-Tg (MMTV-PyVmT) mice, transplanted into syngeneic FVB/N hosts and characterized. Met1 maintains a stable morphological and biological phenotype after multiple rounds of *in vitro* culture and *in vivo* transplantation. The Met1 line tumors exhibit invasive growth and 100% metastasis when transplanted into the mammary fat pads of FVB/N females. The DNA content and gene expression levels of Met1 cells are stable over multiple generations (Borowsky et al., 2005). For this study, Met1 cells were grown in DME (Invitrogen, Grand Island, NY) supplemented with 10% (v/v) fetal bovine serum (Invitrogen) and 100U/mL penicillin-streptomycin (Invitrogen). Cultures that reached at least 70% confluence were trypsinized, washed 3 times with phosphate-buffered saline (Invitrogen) and counted.

### Mammary gland dissociation

The media used for mammary gland dissociation was serum-free 1:1 mixture by volume of Dulbecco's Modified Eagle's Medium: Ham's Nutrient Mixture F12 (DMEM/Ham's F12, Invitrogen, Carlsbad, CA, USA) buffered with HEPES (Invitrogen) supplemented with 0.5 mg/ml Penicillin/Streptomycin (Invitrogen), 2% bovine serum albumin fraction V (Invitrogen), 5 μg/mL insulin, 10 ng/mL cholera toxin (Sigma Aldrich), and 3 mg/mL collagenase (Worthington Biochemical Corp., Lakewood, NJ, USA). Normal mammary gland tissues were obtained from FVB/NJ female mice. MINO tissues were obtained from FVB/NJ mice that had been previously transplanted with MIN (1 mm × 1 mm) tissue. The MINO tissue was removed 4 weeks after transplantation as described (Maglione et al., 2001). PyVmT tumors were from transgenic PyVmT females that had developed mammary tumors. To dissociate tissues, mice were anesthetized using Nembutal (60 mg/kg) and selected tissues were removed from live, anesthetized animals. Following tissue removal, mice were euthanized using an overdose of Nembutal (120 mg/kg). Tissues were mechanically minced with a McIlwain tissue chopper (Mickle Laboratory Engineering, Guildford, UK) with enough serum-free digestion reagent to allow for complete mixing of the tissue. This mixture was digested in a sterile 50 mL tube with gentle agitation for 16 h at room temperature. The resulting suspension was centrifuged at 80 × *g* for 4 min to separate the fatty layer including the supernatant, and the pellet was rinsed with a 1:1 mixture of DMEM/Ham's F12 to eliminate digestive enzymes. To obtain a single cell suspension, the rinsed pellet was first broken up by gently pipetting and then incubated with 0.25% trypsin/EDTA (Invitrogen) at 37°C for 1 to 2 min. The level of dissociation in the suspension was checked under a microscope and then 0.1 mg/mL DNase I was added and the sample was incubated for a further 5 min at 37°C. A volume of DMEM/Ham's F12 (supplemented with 10% fetal bovine serum) equal to the volume of the single cell suspension was then added to stop trypsin activity. Any remaining cell clumps were removed by filtration through a 40 μm cell strainer (BD Biosciences, San Jose, CA).

### Coverslip preparation

Round coverslips (BD biosciences, San Jose, CA) were coated with 5 μg/cm^2^ of laminin and incubated in 6-well dishes for 30 min at 37°C. The remaining material was aspirated after incubation, and 5000 cells were plated per coverslip, 18–24 h prior to measuring the pH_i_.

### Intracellular pH measurement

The pH_i_ was measured using the fluorescent ratio dye 2′,7′-bis(2-carboxyethyl)-5(6)-carboxyfluorescein (BCECF; Molecular Probes). Cells on coverslips were loaded with 2.5 μM of BCECF-AM in HEPES-buffered Ringer's solution (HR) for 30 min at 37°C, under 0% CO_2_. Coverslips were washed three times in HR, incubated for 30 min in HR at 37°C, under 0% CO_2_, and then transferred to a polystyrene chamber that permitted continuous superfusion of the solution. Cells were maintained at 37°C in the chamber and the fluorescence of BCECF was measured at an emission wavelength of 535 nm using the optimized excitation wavelengths of 490 and 440 nm. Wavelength switching was controlled by a DX-1000 optical switch (Solamere Technologies, Salt Lake City, UT). Images were captured with a Stanford Photonics camera, and quantitative analysis was performed using OpenLab Image analysis software (Perkin Elmer, Waltham, MA). Five-point standard curves were generated for BCECF using high-K^+^ solutions of known pH in conjunction with 10 μM nigericin (Sigma). Complete calibration curves were constructed over the pH range of 6.2–8.2. Thereafter a single calibration point was measured at the end of each experiment as described by Boyarsky et al. (1988a). Fixed buffer capacity (ß in mM/pH) for normal mammary epithelial cells (nMECs), Met1, and MINO cells were also measured on separate coverslips as previously described Boyarsky et al. (1988a). The equations used to calculate buffer capacity are = −52.95pH_i_ + 400 for nMEC, = −38.55pH_i_ + 293 for Met1, and = −29.01pH_i_ + 227 for MINO. At pH_i_ = 7 these correspond to buffer capacities of 29, 24, and 23.5 mM/pH for nMEC, Met1, and MINO, respectively, and they are not significantly different.

Solutions: The standard HR was composed of the following concentration in mM: 133 NaCl, 4.75 KCl, 20 HEPES, 1.25 MgCl_2_, 1.82 CaCl_2_, 11.1 glucose, and 8 NaOH, adjusted to pH 7.4 at 37°C with NaOH or HCl. High-K^+^ calibration solutions contained, in mM: 140 KCl, 20 HEPES, 1.25 MgCl_2_, 0.5 CaCl_2_, and 11.1 glucose, using KOH or HCl to adjust pH. In NH_3_/${\text{NH}}_{4}^{+}$-containing solutions, NaCl was replaced with NH_4_Cl at a ratio of 1:2 milliequivalents.

### Washout experiments

For sodium washout experiments, cells growing on laminin-coated slides were loaded with BCECF-AM and superfused with HR buffer for 2 min and then superfused with a Na^+^-free solution. The Na^+^-free solution was the same as standard HR solution but with an equimolar substitution of NaCl by N-methyl-D-glucamine-Cl. For ammonium washout experiments, cells growing on laminin coated slides were loaded with BCECF and superfused with HR buffer for 2 min. Intracellular acid loading was induced by superfusing cells with HR+20 mM NH_4_Cl solution followed by superfusion with a Na free solution. The BCECF signal was calibrated as described above (See Figure S1 in Supplementary Material).

### Glucose uptake assay

Normal mammary epithelium (for nMEC) and MINO were dissociated and single cell suspensions were prepared and counted prior to measurement of glucose uptake. Met1 cells were trypsinized and single cell suspensions were also prepared. Glucose uptake per cell was measured using the fluorescent glucose analog 2-[N-(7-nitrobenz-2-oxa-1,3-diazol-4-yl) amino]-2-deoxy-D-glucose (2-NBDG). Cells were incubated with 200 μM 2-NBDG at 37°C with 5% CO_2_ for 20 min. Cells were washed twice with Dulbecco's phosphate buffered saline (PBS, Invitrogen) and prepared for flow cytometry. 2-NBDG treated suspensions of cells were stained with 4′,6-diamidino-2-phenylindole (DAPI), CD45, TER119 and CD31 (Semerad et al., 2005; Christopher and Link, 2008) and sorted on a FACS Aria flow cytometer (BD Biosciences). FlowJo software (Tree Star, Inc., Ashland, OR) was used to analyze the data.

### Histology and immunohistochemistry

Paraffin sections (4 μm) were stained with Mayer's hematoxylin and eosin or immunostained as described previously (Maglione et al., 2004). The following primary antibodies were used with the Vectastain ABC Elite Kit (Vector Laboratories, Burlingame, CA): guinea pig anti-cytokeratin 8/18 (guinea pig anti-CK8/18; 1:1000; RDI- Research Diagnostics Inc., Concord, MA), rabbit anti-glucose transporter 1 (rabbit anti-GLUT1; 1:100; Dako, Carpinteria, CA, USA), and rabbit anti-carbonic anhydrase IX (rabbit anti-CAIX; 1:250; Novus Biologicals). The Dako ARK kit (Dako) was used for immunohistochemistry with mouse anti-smooth muscle actin (SMA; 1:100; Sigma, St. Louis, MO) antibody. Images of slides were captured using 20 × and 40 × objectives on an AxioScope microscope (Carl Zeiss Inc., Thornwood, NY) with AxioCam camera and processed using Adobe Photoshop 7.0 (Adobe Systems, Inc., San Jose, CA, USA) software.

### Statistical analysis

Unless otherwise stated analysis of variance for repeated measures was used to test for differences in the data. When differences were found, the Student-Newman-Keuls multiple comparison test was used *post-hoc* to determine which groups were different with *P* < 0.05 chosen as indicative of significant differences. Welch's *t*-test was used to compare individual pHi values from the ammonium washout experiments. Linear regression was chosen to describe the rate of the acidification followed by an analysis of covariance (ANCOVA) which was used to compare the slopes of the fitted lines. Results are reported as mean ± standard error.

## Results

### MINO precancer has variable expression of GLUT1 and CAIX compared with MINO tumor

**Figure 1:**
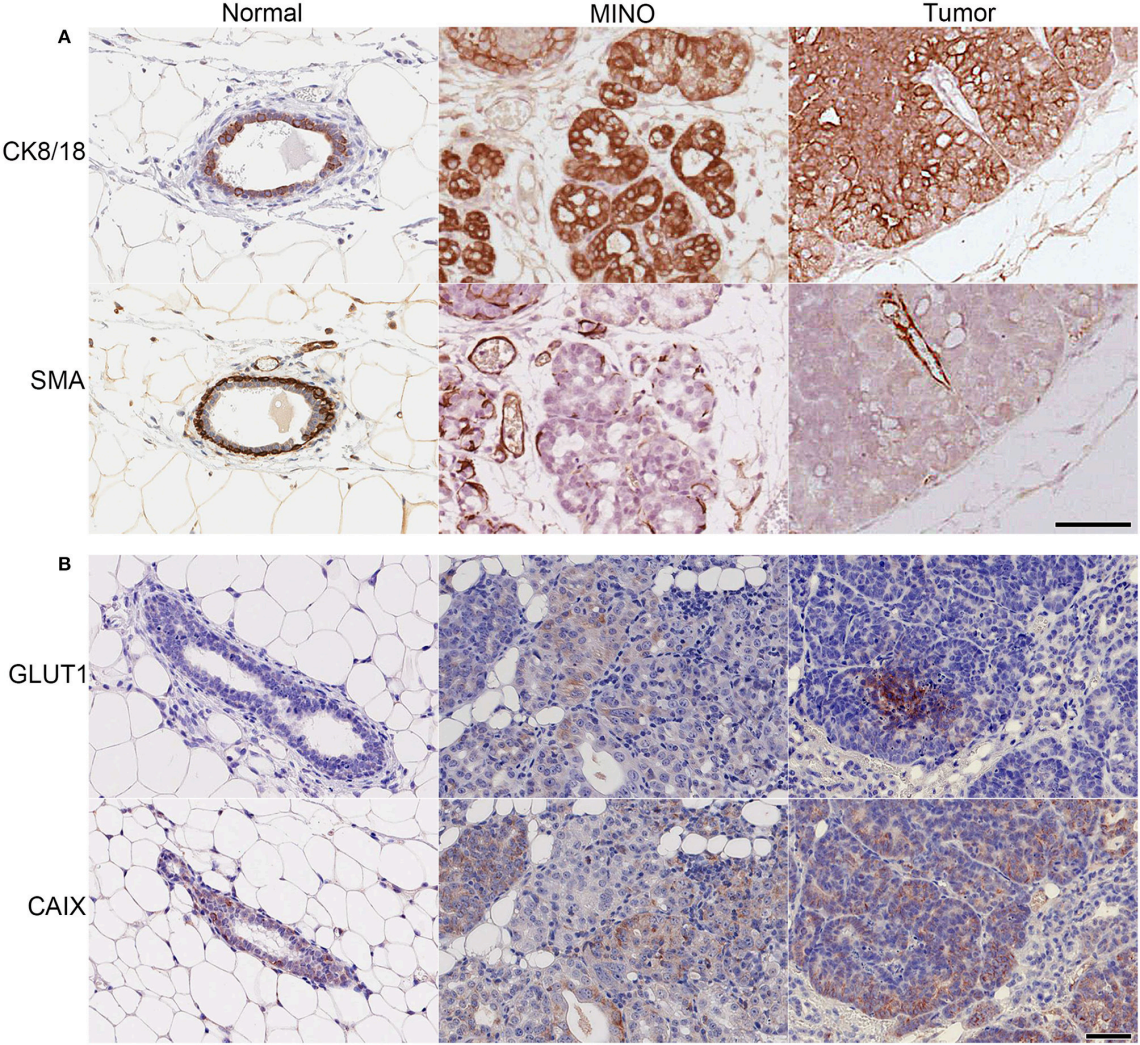
**MINO tumor cells originate from ductal (CK8/18^+^), not myoepithelial (SMA^+^) cells and have high expression of GLUT1 and CAIX compared with normal mammary gland or MINO tissues**. Immunohistochemical staining of normal, MINO, and MINO tumor tissue for **(A)** the structural proteins cytokeratin 8/18 (CK 8/18) and smooth muscle actin (SMA) and for **(B)** glucose uptake, glucose transporter 1 (GLUT1), and hypoxia, carbonic anhydrase 9 (CAIX). The scale bars on the lower right image in **(A)** and **(B)** are 50 μm in length and are representative for each of the other images in the panels.

MINO precancer and the invasive carcinomas arising from MINO (MINO tumor) with CK8/18, a marker of luminal epithelial cells and SMA, a marker for basal myoepithelial cells showed organized multi-lineage differentiation in the MINO precancer whereas invasive carcinoma cells have uniform cellular immunoreactivity for CK8/18 with SMA only in the smooth muscle cells associated with blood vessels (Figure 1). Next we stained MINO and MINO tumor for the expression of GLUT1 and CAIX. Glucose uptake via the glucose transport proteins (GLUT1-9) is a rate-limiting step in this glycolysis (Adekola et al., 2012). GLUT1 is thought to be the main glucose transporter in breast cancer (Adekola et al., 2012; Szablewski, 2013) and CAIX is a marker of hypoxia linked to carcinogenesis (van Brussel et al., 2013). With respect to GLUT1, there was no protein expression in the normal tissue, weak expression in some cells in the MINO tissue, and strong, localized expression in the tumor. A few cells expressed CAIX in the normal tissue, in contrast to the tumor, which expressed diffuse CAIX. The presence of specific subpopulations of cells expressing CAIX in the MINO suggests metabolic heterogeneity within the MINO.

### Intracellular glucose requirements are heterogeneous in MINO cells compared with normal and cancer cells

**Figure 2:**
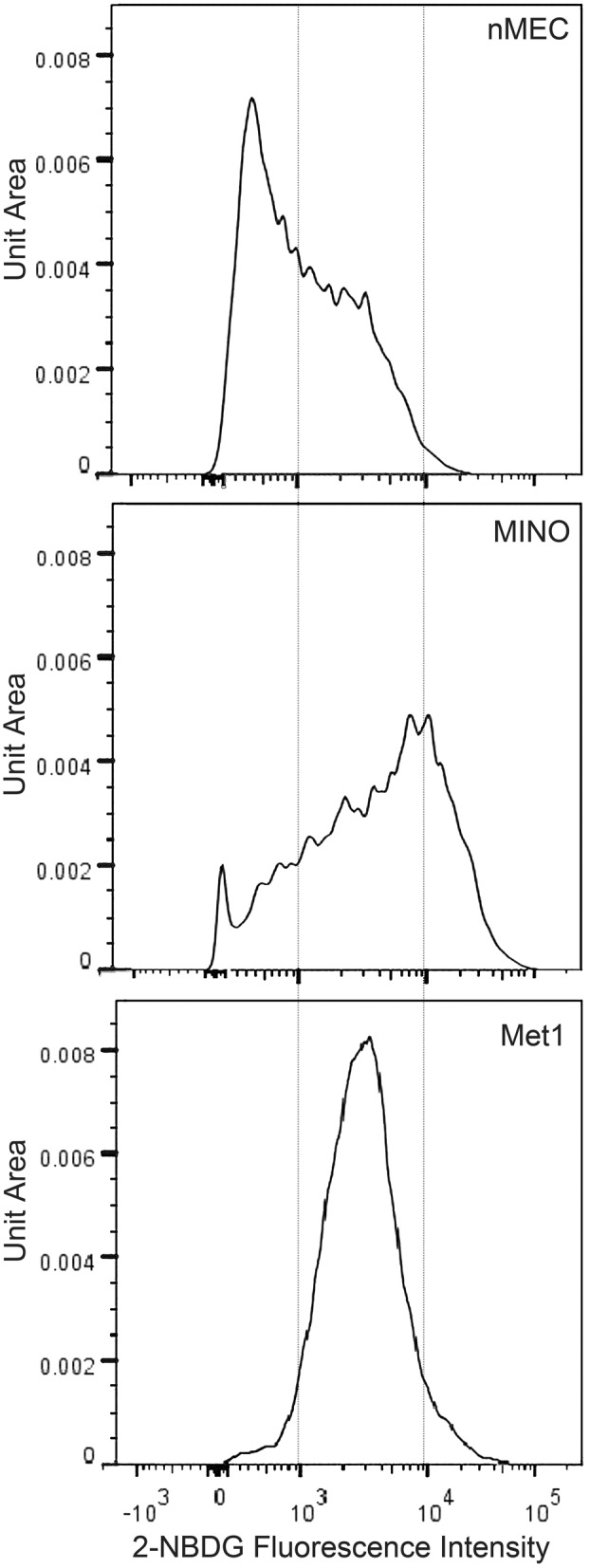
**Qualitative differences in glucose uptake in normal (nMEC), precancer (MINO) and cancer (MET1)**. Glucose uptake per cell was measured using a fluorescent glucose analog dye (2-NBDG) and flow cytometry. The histograms of the three samples (nMEC, MINO, and Met1 cells) were normalized to unit area in order to compare the distribution profiles. In this plot, the horizontal axis shows the intensity of the 2-NBDG signal (divided into 256 bins) and the vertical axis represents the percent of cells (events) that fall within each bin. The histogram plots for the nMEC and MINO represent the populations after dead and hematopoietic cell depletion.

The heterogeneity of GLUT1 levels observed in MINO and MINO tumor led to the hypothesis that cells within the MINO would exhibit heterogeneous glucose uptake. The non-radioactive fluorescent glucose analog dye, 2-NBDG, was used to measure glucose uptake in single nMEC, MINO, and Met1 cancer cells (Borowsky et al., 2005). 2-NBDG imaging has been validated in live cells *in vitro* as a sensitive probe for monitoring glucose metabolism in malignant cells (O'Neil et al., 2005; Yamada et al., 2007). Figure 2 shows qualitative differences in glucose uptake in the nMEC, MINO and Met1 cells. In order to compare the distribution of glucose uptake in nMEC, MINO and Met1 cells, histograms of fluorescence intensity of 2-NBDG for each individual group of cells were normalized to unit area. The vertical axis represents the normalized number of cells with a given intensity of the 2-NBDG signal. The histogram plots for the nMEC and MINO represent the populations after dead cells and hematopoietic cells were depleted. The nMEC cells displayed a roughly normal distribution of glucose uptake with a 2-NBDG signal that peaked below 10^3^ units of fluorescence intensity, indicating low glucose uptake (Figure 2). Met1 cancer cells displayed a normal distribution of glucose uptake with the 2-NBDG signal peaking above 10^3^ units of fluorescence intensity indicating that the majority of Met1 cells had an increased glucose uptake. Cells within the MINO population displayed a wider distribution, a wider range of 2-NBDG fluorescence intensities indicating a wider range of glucose uptake levels. The shape and wider distribution of the curve suggest that there is a higher level of heterogeneity in glucose uptake in the MINO population than in the Met1 or nMEC populations. Furthermore, the shape of the distribution suggests that there may be two or more overlapping populations with different (higher and lower) average glucose uptakes. This data suggests that there is a coexistence of metabolically different cell populations within the MINO.

### Cancer cells (Met1, PyVmT) have a higher baseline NHE1 activity and a more alkaline baseline intracellular pH compared with normal mammary epithelial cells

To further investigate the metabolic profiles of MINO cells compared with tumor cells we measured intracellular pH in a bicarbonate-free system (Boyarsky et al., 1988a,b). Dissociated, single nMEC, MINO, and Met1 cells were loaded with BCECF and perfused with solutions that either induced acidification of the cytoplasm (ammonium washout experiment, see Figure S1) or inhibited cell membrane proton transport (sodium washout experiment).

**Figure 3:**
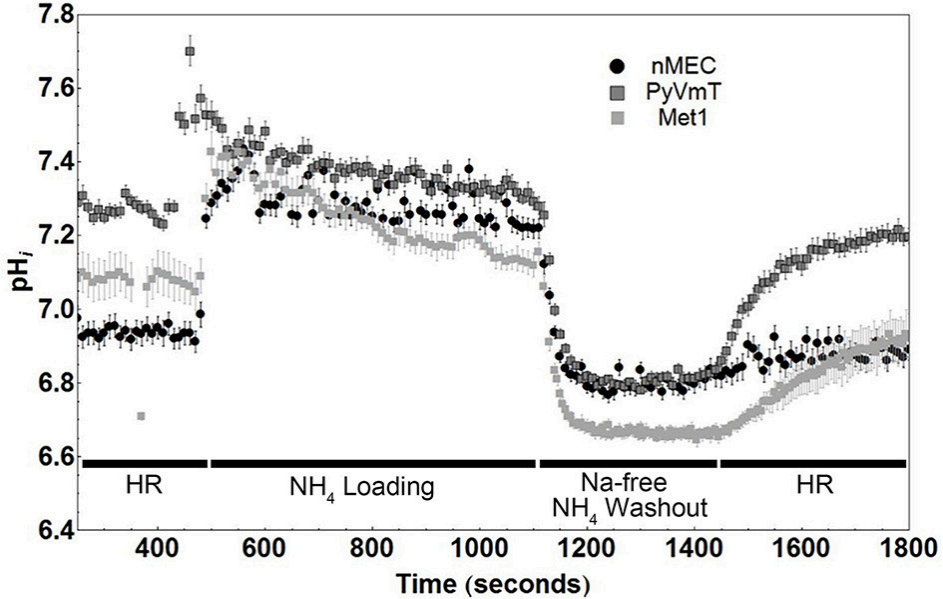
**Cancer cells (PyVmT tumor and Met1) have higher baseline intracellular pH and recover faster from the acid load than normal mammary epithelial cells (nMEC)**. Baseline pH_i_ and rates of recovery of pH_i_ when cells are challenged with an intracellular acid load (ammonium washout experiment). Markers on the plot represent the average and standard error of each pH_i_ measurement. Number of cells measured: nMEC = 11, Met1 = 5, PyVmT = 10. Mean baseline pHi values were calculated between 250 and 400 sec: Met1 = 7.06 ± 0.05, PyVmT = 7.28 ± 0.02, nMEC = 6.94 ± 0.03. The baseline pHi values of both cancer cell types (PyVmT and Met1) are significantly (*P* < 0.05) more alkaline compared with nMEC cells. HR; HEPES Ringer's Solution.

In a bicarbonate-free system, the main transporter responsible for extruding protons from the cell is NHE1, and NHE1 activity has been found in the earliest steps of cancer progression (Cardone et al., 2005). NHE1 activity is inhibited when cells are superfused with a sodium free buffer. This inhibition, under baseline conditions, allows for the measurement of baseline proton production and otherwise concomitant efflux via NHE1. We performed ammonium washout experiments on nMEC, Met1, and PyVmT tumor-dissociated cells. Figure 3 is a representative figure of the data from these experiments. We found that both types of cancer cells, Met1 and PyVmT, had baseline intracellular pH values (7.06 ± 0.05 and 7.28 ± 0.02, respectively) that were significantly (*P* < 0.05) more alkaline compared with nMEC (6.94 ± 0.03). Furthermore, after intracellular acidification (by ammonium chloride loading and washout), both types of cancer cells recovered faster than the nMEC, suggesting an increase in NHE1 activity (Figure 3). Linear regression followed by analysis of covariance (ANCOVA) for the representative data of the pH_i_ during the recovery phase (1400–1800 s) from nMEC, Met1, and PyVmT cells showed that the slope of the best-fit lines for both cancer cell types (Met1 and PyVmT) were significantly different (*P* < 0.001) compared with the slope of the best-fit line for nMEC (Met1 slope = 7.678 × 10^−4^ pH_i_ sec^−1^, PyVmT slope = 1.008 × 10^−3^ pH_i_ sec^−1^, nMEC slope = 1.721 × 10^−4^ pH_i_ sec^−1^, Figure S2). Multiplying buffer capacity (ß) by the rate of change in pH_i_ gives the rate of efflux via NHE1. For the representative data in Figure 3 the initial flux rates during pH_i_ recovery were 4.75 for PyVmT, 2.3 for Met1, and 1.5 mM/min for nMEC (flux for Met1 and nMEC were calculated using ß values calculated as described above. Buffer capacity for Met1 was used to calculate the flux for PyVmT).

### MINO tissue is metabolically heterogeneous

**Figure 4:**
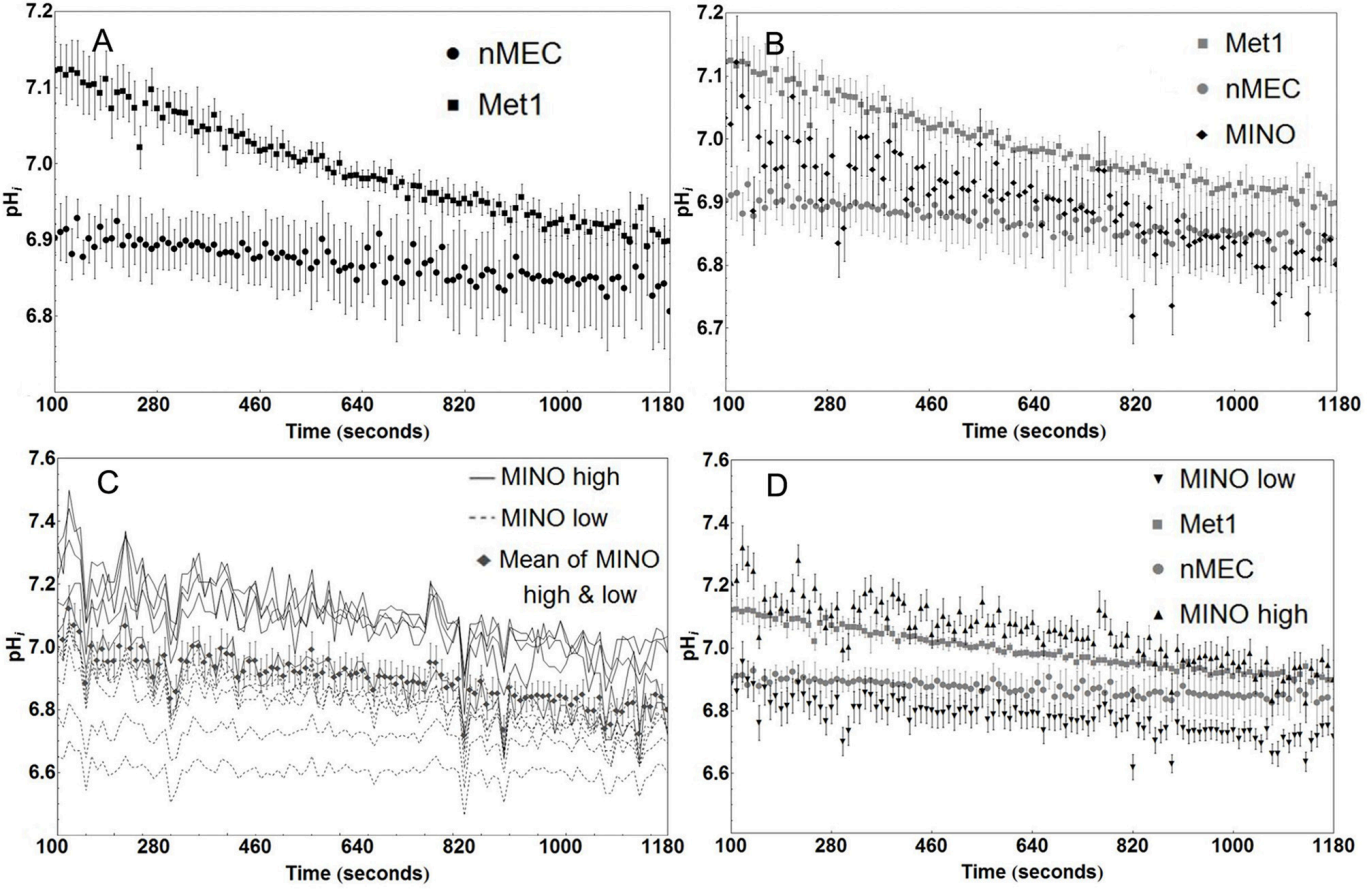
**MINO cells display metabolic heterogeneity compared with Met1 and nMEC**. Cancer cells (Met1, *n* = 6 coverslips) have a higher rate of intracellular acidification than normal mammary epithelial cells (nMEC, *n* = 3 coverslips) as measured by sodium washout experiments. Mean pHi values were significantly different in more than 98% percent of the measured time points prior to but none after 430 s (Welch's *t*-test, *P* < 0.05). **(A)** The mean rate of acidification in MINO cells (*n* = 11 cells) falls nominally between the nMEC and Met1 rates of acidification, without significant difference **(B)**. Baseline pHi for individual MINO cells divide into two groups that we called MINO high (*n* = 6 cells) and MINO low (*n* = 5 cells) **(C)**. MINO high cells share similar baseline pHi as Met1 cells, MINO low cells share similar baseline pHi as nMEC cells. The separation of MINO high and low data reflects the Met1 and nMEC attributes, respectively. **(D)** Markers on the plot represent the average and standard error for pHi at each time point.

Met1 cancer cells have a higher rate of proton production than nMEC (Figure 4A), as measured by the rate of intracellular acidification during the sodium washout experiments where proton extrusion was blocked by inhibiting NHE1 in nominally bicarb-free medium. Individual pHi values of nMEC and Met1 cells were significantly different in more than 98% percent of the measured time points in the first 430 s (Welch's *t*-test, *P* < 0.05). Linear regression analysis for nMEC and Met1 individual pHi values followed by analysis of covariance (ANCOVA) showed significant difference (*P* < 0.001), between the slopes of the best-fit lines (Met1 slope = −2.037 × 10^−4^ pH_i_ sec^−1^, nMEC slope = −0.617 × 10^−4^ pH_i_ sec^−1^, Figure S3). The more negative slope from the Met1 cell data suggests more rapid proton production compared with nMEC. There was large variation in the mean rate of change in pHi in MINO cells (Figure 4B). Based on baseline pH_i_, individual MINO cells (Figure 4C) were separated into two groups that we designated MINO high and MINO low (Figure 4D). The MINO high group of cells had higher baseline pH_i_ and more rapid fall in pHi (ΔpH_i_ = −2.74 × 10^−4^ sec^−1^) than the Met1 (ΔpH_i_ = −0.037 × 10^−4^ sec^−1^), the MINO low group (ΔpH_i_ = −1.471 × 10^−4^ sec^−1^) or the nMEC (ΔpH_i_ = −0.617 × 10^−4^ sec^−1^). The slopes of the best-fit lines from the MINO low and MINO high pHi values were, however, not significantly different.

**Figure 5:**
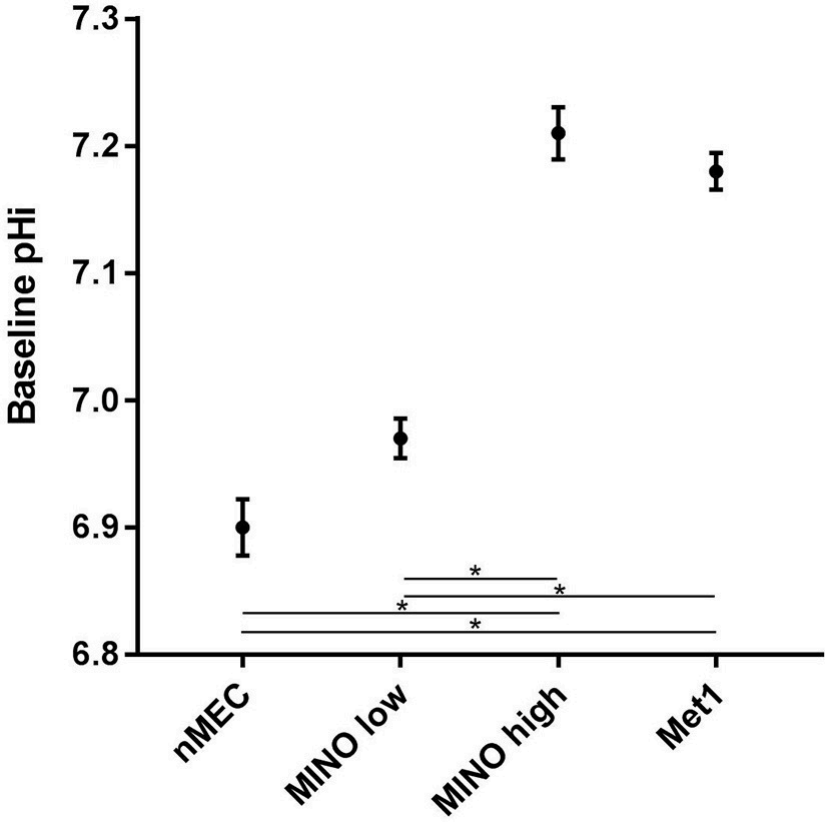
**Metabolic heterogeneity exists within the MINO, some cells are more cancer-like in their metabolism while others retain a more normal-like metabolism**. Mean baseline pH_i_ and standard error are shown for Met1 [7.18 ± 0.01; *n* = 8 coverslips (145 cells)], nMEC [6.9 ± 0.02; *n* = 9 coverslips (120 cells)], MINO high [7.21 ± 0.02; *n* = 4 coverslips (9 cells)] and MINO low [6.97 ± 0.02; *n* = 7 coverslips (59 cells)]. Means were calculated from the first 200 s of measurement. Analysis of variance for repeated measures demonstrated differences in baseline pH_i_. The Student-Newman-Keuls multiple comparison test was used *post-hoc* to determine which groups were different with *P* < 0.05 chosen as indicative of significant differences. ^*^P < 0.05.

Under baseline conditions (Figure 5), the mean pH_i_ of Met1 cells (7.18 ± 0.01; *n* = 8 coverslips (145 cells)) was significantly (*P* < 0.05) more alkaline than that of nMEC (6.9 ± 0.02; *n* = 9 coverslips (120 cells)). MINO high (7.21 ± 0.02; *n* = 4 coverslips (9 cells)) resting pHi was significantly (*P* < 0.05) more alkaline than MINO low (6.97 ± 0.02; *n* = 7 coverslips (59 cells)). The baseline pH_i_ for MINO high cells was also significantly different from the baseline pH_i_ for nMEC and the baseline pH_i_ for MINO low was significantly different from that of Met1 cells.

## Discussion

The standard of care for patients diagnosed with DCIS is eradication treatment by surgery and/or radiation. A better stratification of risk for progression could dramatically reduce overtreatment in the clinic. Using a mouse model of DCIS, the MINO mouse model, we conducted a series of experiments to define the relationship between DCIS and invasive cancer. Here we investigated the hypothesis that a subset of cells within the MINO precancer has a cancer-like “Warburgian” (Warburg, 1956) glucose uptake and pH regulation profile. We first asked whether MINO precancer and tumor cells were heterogeneous in their levels of markers of glucose transport and hypoxia. We showed the presence of a subset of GLUT1 and CAIX positively stained MINO cells indicating that MINO tissue might be metabolically heterogeneous (Figure 1). CAIX is a hypoxia inducible protein that also regulates cell pH (Airley et al., 2003). Increased expression of both GLUT1 and CAIX in breast cancer has been found to correlate to metastasis, invasion, and poor survival outcomes (Pinheiro et al., 2011; Lock et al., 2012). 2-NBDG uptake in individual nMEC, MINO precancer, and Met1 cancer cells (Figure 2) was different, and the wide range of distribution in the MINO cells suggests that MINO cells are not only microanatomically and functionally heterogeneous (Maglione et al., 2004; Namba et al., 2004, 2006; Damonte et al., 2008) but are also metabolically heterogeneous. FDG-PET imaging studies in the MINO mouse model have previously shown that precancerous tissue has increased uptake of glucose compared with normal mammary epithelium, though less than the invasive cancers (Abbey et al., 2004, 2006). FDG-PET signal per voxel is the product of individual cell uptake and the cell density. Cell density is increased in invasive carcinoma compared with MINO, but individual cell glucose uptake in MINO might also be increased. We have shown here that MINO precancer tissue is composed of populations of individual cells with different glucose uptake and pH_i_ regulation. Our previous work documents that all of the cell types in the MINO are derived from individual precancer “stem-like” cells (Damonte et al., 2008).

To investigate pH regulation within individual cells we measured the pH_i_ with the pH-sensitive dye BCECF using ammonium washout (Figure 3) and sodium washout (Figure 4) experiments. Using single cells from nMEC, PyVmT tumor, and the Met1 cancer cell line we determined that baseline pH_i_ was more alkaline in both tumor cells as compared with normal cells. Moreover, the rate of pH_i_ recovery after NH_4_Cl washout was faster in both types of cancer cell lines, suggesting that cancer cells have higher NHE1 activity. NHE1 is critical in normal mammary branching morphogenesis (Jenkins et al., 2012), helps to maintain an alkaline pH_i_ in cancer cells (McLean et al., 2000), and is involved in the early events leading to the malignant transformation and G2/M entry of NIH3T3 mouse fibroblasts (Reshkin et al., 2000; Putney and Barber, 2003) as well as for human cancer cell polarization and invasion (Lagana et al., 2000; Reshkin et al., 2000; Paradiso et al., 2004).

Next we measured intracellular proton production rates by superfusing cells with sodium ion-free buffer thereby inhibiting NHE1 activity. The Met1 cells had a higher mean rate of fall in pH_i_ suggesting a higher mean rate of proton production than the nMEC (Figure 4A) and, interestingly, the mean MINO acidification rate fell roughly between the acidification rates of the nMEC and Met1 cells (Figure 4B). An examination of the individual MINO baseline pH values showed that MINO cells could be divided into a MINO high group and a MINO low group (Figure 4C). When we averaged and plotted the MINO high and MINO low groups separately we found both MINO groups had a nominally greater acidification rate than Met1 cells and MINO high cells had a nominally greater acidification rate compared with MINO low cells (Figure 4D). Finally, the mean baseline pH_i_ for Met1 and MINO high were more similar to each other than to nMEC and MINO low and vice versa (Figure 5). Ideally it would be advantageous to be able to identify the cell type with respect to luminal and basal markers, however, we have not yet been able to immunophenotype the cells in the flow chambers. Nevertheless, these experiments demonstrate distinct populations within the MINO of cells with cancer-like and normal-like metabolisms. These data collectively confirmed our hypothesis that a subset of cells with altered metabolism (higher glucose uptake and proton extrusion rate) exists within the MINO.

Literature suggests that the transition to glycolysis and the acidification of the microenvironment plays a significant role in cancer progression. This, together with our finding that precancer tissue shows heterogeneity in NHE1 activity and glucose uptake raises the question of the role of this subset of cells in the progression from precancer to invasive cancer. The frequency and location of these cells in the precancer may correlate with progression rates. Moreover, this raises an interesting mechanistic question about the role of pH micro-gradients as a component of the cancer and precancer microenvironment.

## Author contributions

RL participated in the coordination and interpretation of the results, performed statistical analysis and drafted the manuscript. NH helped with interpretation of data, drafting and critically revising the manuscript. PD participated in the design of the study, prepared tissues for analysis and helped carry out data acquisition. HM and ZP helped with interpretation of data, statistical analysis, and drafting and critically revising the manuscript. CP participated in data acquisition and interpretation of the pH experiments. AK was responsible for the technical aspects as well as interpretation of immunohistochemistry. AG was responsible for carrying out and interpreting glucose uptake studies. SA participated in drafting the manuscript, study design, data acquisition and coordination, interpretation of results and statistical analysis. PC provided expertise in the design and interpretation of the results. AB conceived and designed the study, coordinated and interpreted all data, participated in drafting the manuscript, and revised it critically.

## Funding

Funding was provided by NIH U01 CA014582 and K26 RR024037.

### Conflict of interest statement

The authors declare that the research was conducted in the absence of any commercial or financial relationships that could be construed as a potential conflict of interest.

## Supplementary material

Figure S1 **Cartoon explaining how the NH_4_Cl washout protocol is used to measure pH_i_ regulation after acidification**. When NH_4_Cl is added to the superfusate, the concentrations of NH_3_ in the intra- and extracellular fluids are assumed to be equivalent and, according to the isohydric principle, fixed intracellular buffers are considered to be in equilibrium with NH_3_+H^+^ < >${\text{NH}}_{4}^{+}$. In step 1, NH_3_ enters the cell as a gas to raise pH_i_. In step 2 pH_i_ falls as ${\text{NH}}_{4}^{+}$ enters the cell via cation transporters. In step 3, NH_4_Cl is washed out of the extracellular space with Na-free media after which NH_3_ gas exits the cell leaving excess protons behind and acidifying the cell. In step 4 normal Na is replaced in the superfusate and Na-dependent transporters regulate pH_i_.

Figure S2 **Linear regression followed by analysis of covariance (ANCOVA) for the representative data of the pHi during the recovery phase (1400–1800 s) of the ammonium washout experiment in nMEC, Met1, and PyVmT cells showed that the slope of the best-fit lines for both cancer cell types (Met1 and PyVm) were significantly different (*P* < 0.001) compared with the slope of the best-fit line for nMEC (Met1 slope = 7.678 × 10^−4^ pH_i_ sec^−1^, PyVmT slope = 1.008 × 10^−3^ pH_i_ sec^−1^, nMEC slope = 1.721 × 10^−4^ pH_i_ sec^−1^)**.

Figure S3 **Met1 cancer cells have a higher rate of intracellular acidification than nMEC cells during sodium washout experiments where proton extrusion is blocked by NHE1 inhibition**. Linear regression analysis for nMEC and Met1 individual pHi values followed by analysis of covariance (ANCOVA) showed significant difference (*P* < 0.001), between the slopes of the best-fit lines (Met1 slope = 2.037 × 10^−4^ pH_i_ sec^−1^, nMEC slope = 0.617 × 10^−4^ pH_i_ sec^−1^). The more negative slope from the Met1 cell data suggests more rapid proton production compared with nMEC.

## Abbreviations

Mino: Mammary Intraepithelial Neoplasia Outgrowth
Nmec: Normal Mammary Epithelial Cells.
