# Supplementary figures and images for: Glucose Uptake and Intracellular pH in a Mouse Model of Ductal Carcinoma In situ (DCIS) Suggests Metabolic Heterogeneity
Source: Front Cell Dev Biol. 2016 Aug 31;4:93. doi: 10.3389/fcell.2016.00093 (PMC5005977; doi:10.3389/fcell.2016.00093)

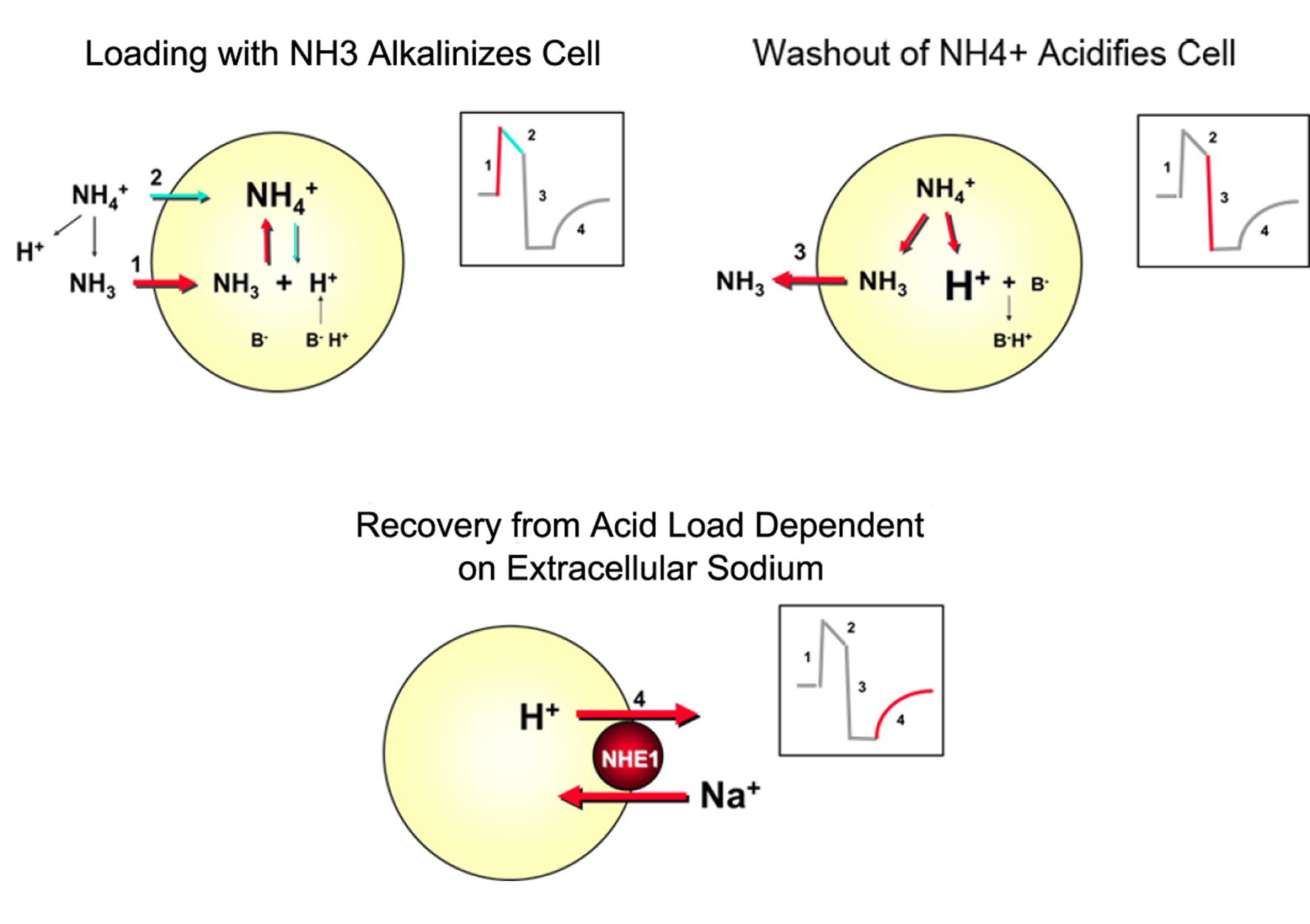

Supplement: Figure S1 — Cartoon explaining how the NH4Cl washout protocol is used to measure pHi regulation after acidification. When NH4Cl is added to the superfusate, the concentrations of NH3 in the intra- and extracellular fluids are assumed to be equivalent and, according to the isohydric principle, fixed intracellular buffers are considered to be in equilibrium with NH3+H+ < >NH4+. In step 1, NH3 enters the cell as a gas to raise pHi. In step 2 pHi falls as NH4+ enters the cell via cation transporters. In step 3, NH4Cl is washed out of the extracellular space with Na-free media after which NH3 gas exits the cell leaving excess protons behind and acidifying the cell. In step 4 normal Na is replaced in the superfusate and Na-dependent transporters regulate pHi. [file Image1.TIF]

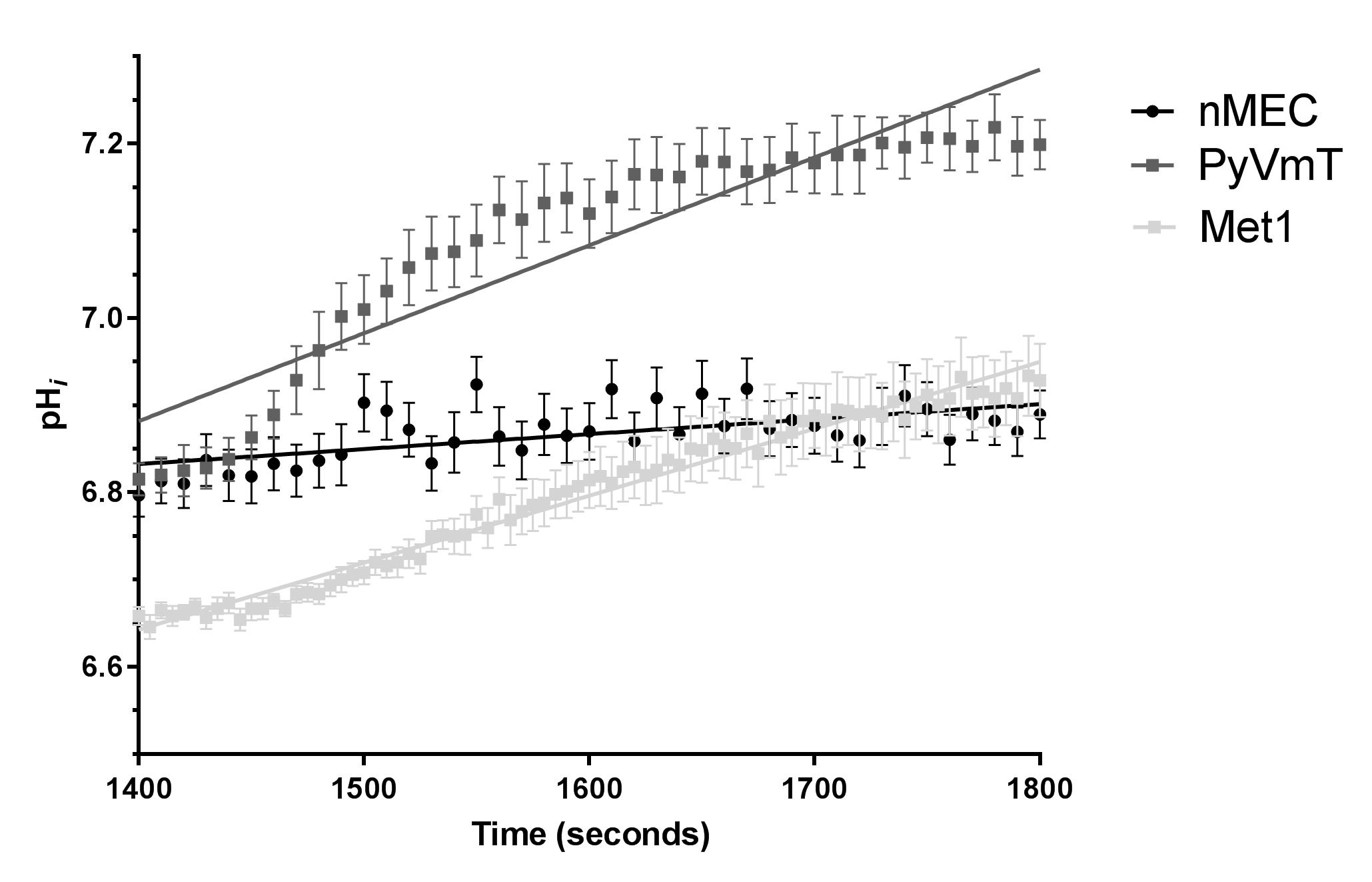

Supplement: Figure S2 — Linear regression followed by analysis of covariance (ANCOVA) for the representative data of the pHi during the recovery phase (1400–1800 s) of the ammonium washout experiment in nMEC, Met1, and PyVmT cells showed that the slope of the best-fit lines for both cancer cell types (Met1 and PyVm) were significantly different (P < 0.001) compared with the slope of the best-fit line for nMEC (Met1 slope = 7.678 × 10^−4^ pHi sec−1, PyVmT slope = 1.008 × 10^−3^ pHi sec−1, nMEC slope = 1.721 × 10^−4^ pHi sec−1). [file Image2.JPEG]

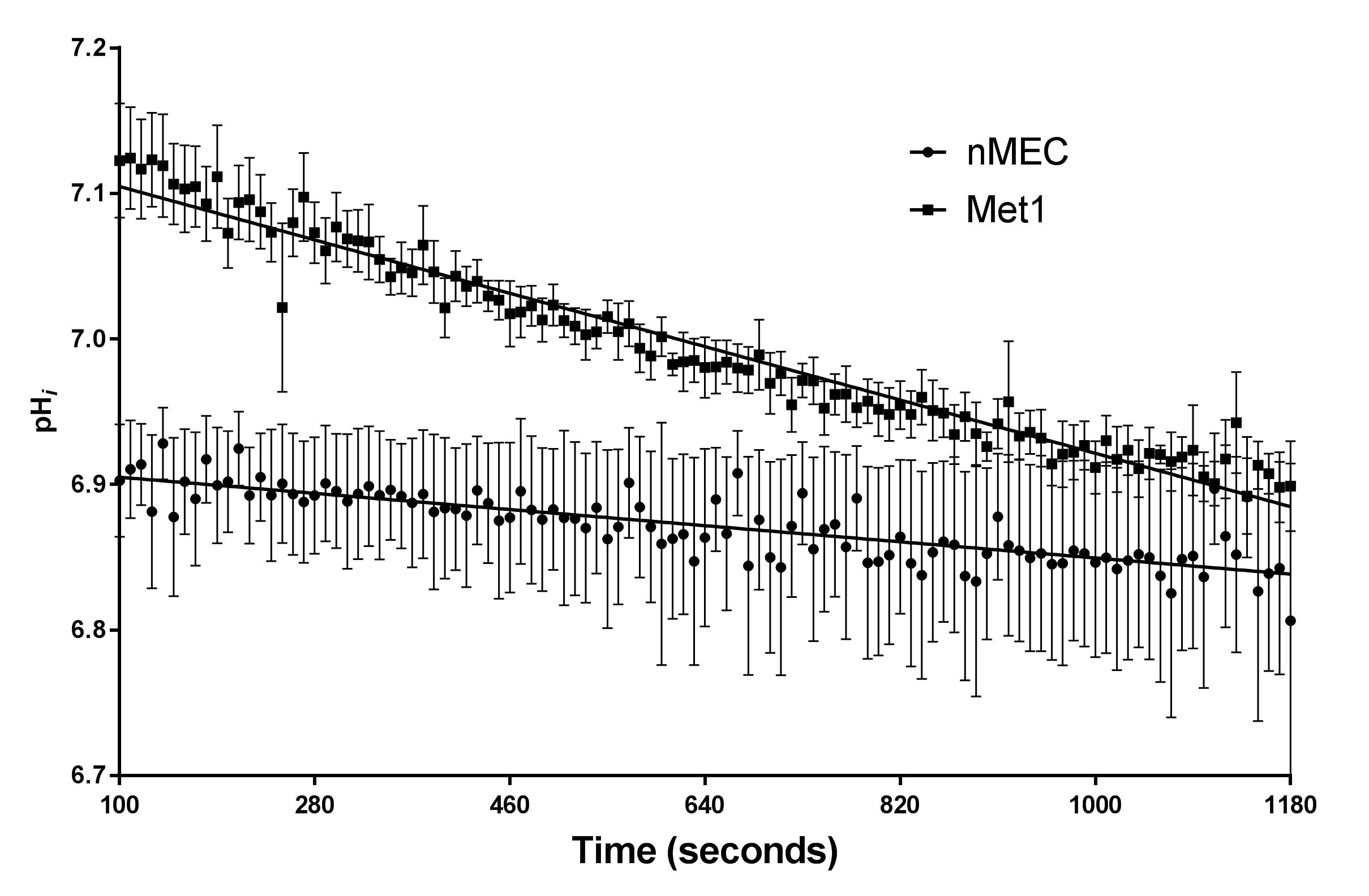

Supplement: Figure S3 — Met1 cancer cells have a higher rate of intracellular acidification than nMEC cells during sodium washout experiments where proton extrusion is blocked by NHE1 inhibition. Linear regression analysis for nMEC and Met1 individual pHi values followed by analysis of covariance (ANCOVA) showed significant difference (P < 0.001), between the slopes of the best-fit lines (Met1 slope = 2.037 × 10^−4^ pHi sec−1, nMEC slope = 0.617 × 10^−4^ pHi sec−1). The more negative slope from the Met1 cell data suggests more rapid proton production compared with nMEC. [file Image3.JPEG]
